# Supplementary material for: Electroencephalogram-derived pain index for evaluating pain during labor
Source: PeerJ. 2021 Dec 22;9:e12714. doi: 10.7717/peerj.12714 (PMC8710049; doi:10.7717/peerj.12714)
Supplement: Supplemental Information 2 [file peerj-09-12714-s002.docx]

**Pain index calculation**

Using the EEG analysis and calculation software package (Beijing Easymonitor Technology Co., Ltd., Beijing, China) based on a wavelet algorithm, one of the most suitable tools for analyzing EEG, the repeatable and regular changes when pain presents is extracted from brain waves as the characteristic indicator of objective pain.

EEG recorded by scalp electrodes is a comprehensive reflection of the electrophysiological activities of neurons in the brain, it reflects both cortical and subcortical EEG. The physicochemical and electrical characteristics of the potential at any point of the scalp determine the two-compartment analysis method, which is separated into cortical EEG and subcortical EEG. Subsequently, for the decomposed two-compartment EEG signals, we set up four-quadrant EEG analysis arrays, right and left brain and cortical and subcortical regions. EEG data sets are collected to calculate the dynamic and static correlations of each data set and to obtain quantitative data indicators of brain function including pain and analgesia.

In the process of decomposition and reconstruction of wavelet transform in combination with the pattern recognition method, the EEG signal recorded by scalp electrodes is decomposed into cortical EEG and subcortical EEG by wavelet algorithm.

f (t) is transformed by a scale j. The decomposition formula is as follows:

$$\left\{ \begin{aligned} c_{j,k}=\sum_{n} c_{j-1,n}h_{n-2k} \\ d_{j,k}=\sum_{n} d_{j-1,n}g_{n-2k} \end{aligned}(k=0,1,2,\ldots,N-1) \right.$$

The reconstruction formula is as follows:

$$c_{j-1,n}=\sum_{n} c_{j,n}h_{k-2n}+\sum_{n} d_{j,n}g_{k-2n}$$

Among them, $c_{j,k}$ is the scale coefficient, $d_{j,k}$ is the wavelet coefficient, h and g are a pair of orthogonal mirror filter banks, j is the decomposition layer, and N is the discrete sampling points. By decomposing and reconstructing EEG signals, the cortical and subcortical EEG are extracted, and the "two chambers" model of cortical and subcortical EEG is established for interactive analysis. We not only independently analyzed the EEG signals in the cortex and subcortex but also synchronously processed the EEG signals in the cortex and subcortex. Through synthesizing the multidimensional information of the two parts of EEG signals, we comprehensively analyzed the brain's electrical activity.

The decomposed and reconstructed cortical and subcortical EEG signals are discretized to generate vector groups of waveform signals of each lead under specific sampling frequencies, sampling accuracy, and time window. The waveform recognition algorithm and wavelet analysis algorithm are employed to analyze the preprocessed EEG data.

If the f(t) function is a signal in the spatial domain $\left\{ -\infty,+\infty\right\}$, the scaling variable α controls the scaling of the wavelet function, and the translation variable τ controls the translation of the wavelet function. The scale α corresponds to the frequency, while the translation τ corresponds to the time:

$$W_{c}f\left( \tau,a \right)=\frac{1}{\sqrt{a}}\int_{-\infty}^{\infty} f\left( x \right)\Phi\left( \frac{x-\tau}{a} \right)dx$$

Binary discrete wavelet transform is used to calculate the EEG vector. The formula is as follows:

$$W_{2^{J}}f\left( k \right)=<f\left( t \right),\Phi_{2^{j}}\left( k \right)\geq\frac{1}{2^{j}}\int_{R}^{\infty} f\left( t \right)\Phi\left( 2^{-j}t-k \right)dt$$

For the calculation formula of the wavelet frequency domain, the following expression is used:

$$WT_{x}\left( a,\tau\right)=\frac{\sqrt{a}}{2\pi}\int X\left( \omega\right)\Psi\left( a\omega\right)e^{j\omega t}d\omega$$

The algorithm uses a wavelet multi-scale transform and reconstruction algorithm to process specific EEG waveform signals. The generating function is the first derivative of the smoothing function (spline function). A total of 64 points is constructed with scales from 2^0^ to2^6^. The binary wavelet transform is performed:

$$\left( Wf\left( 2^{j},x \right) \right), j\in z$$

Reconstruction function selects partial binary transformation result recovery:

$$f\left( x \right)=\sum Wf\left( 2^{j},x \right)*X2^{j(x)}$$

$X2^{j(x)}$ is the reconstructed wavelet.

For the calculated waveform function f(x), an integral algorithm is used:

$$e={(\int f(x)dx)}^{2}$$

The power $e$ of a specific waveform potential can be obtained.

The peak value of the waveform potential is judged by the maximum and minimum value waveform recognition algorithm, and the obtained time point is used as the weighting factor $a$. $E=a*e$.

The metadata of window EEG calculation can be obtained by a specific combination calculation:

$$\{i\_10,i\_12,i\_15,i\_22,i\_23,i\_24,i\_35,i\_45,i\_48,i\_52,\ldots\ldots\}$$

j is the number of leads, abbreviated as i series index group, where

$\{i\_12,i\_15,i\_23,i\_45,i\_48,\ldots\ldots\}$ is an intermediate variable metadata for cortical EEG signals;

$\{i\_22,i\_35,i\_60,i\_70,\ldots\ldots\}$ is an intermediate variable metadata of subcortical EEG signals;

$\{i\_10,i\_24,i\_52,i\_66\ldots\ldots\}$ is an metadata of intermediate variables.

{i series index group} j = $j\left\{ \mathrm{WLE}\left( i \right),PSD\left( i \right),Gi\left( x \right) \right\}j\&\left\{ a1,a2,\cdot\cdot\cdot\cdot\cdot\cdot\mathrm{an} \right\}j$

Among them,$\left\{ a1,a2,a3,\ldots\ldots,an \right\}$ is the weighted coefficient of the multivariate regression.

By analyzing the decomposition and reconstruction of the above metadata, as well as the relationship between different EEG signal leads, the objective theorem measurement data of different brain states can be obtained by clustering weighting calculation. Quantitative measurements of pain were included, and pain reflects the synergy between the cortex and subcortex. The expression is:

Pain (Pi) =$\left\{ a1,a2\cdot\cdot\cdot\cdot\cdot\cdot\mathrm{an} \right\}\&\left\{ i\_10,i\_15,i\_23,i\_35,i\_45,i\_48,i\_66,i\_70,i\_77 \right\}$.
